# DebtStreamness: an ecological approach to credit flows in interfirm networks

**DOI:** 10.1093/pnasnexus/pgag170

**Published:** 2026-05-18

**Authors:** Anahí Rodríguez-Martínez, Silvia Bartolucci, Francesco Caravelli, Victoria Landaberry, Pierpaolo Vivo, Fabio Caccioli

**Affiliations:** Department of Computer Science, University College London, Gower Street, London WC1E 6EA, United Kingdom; Department of Computer Science, University College London, Gower Street, London WC1E 6EA, United Kingdom; Theoretical Division, Los Alamos National Laboratory, Los Alamos 87545, NM, USA; Financial Stability Department, Banco Central del Uruguay, 777 Diagonal J.P. Fabini., Montevideo 11100, Uruguay; Department of Mathematics, King’s College London, Strand, London WC2R 2LS, United Kingdom; Department of Computer Science, University College London, Gower Street, London WC1E 6EA, United Kingdom; Systemic Risk Centre, London School of Economics and Political Science, Houghton Street, London WC2A 2AE, United Kingdom

**Keywords:** economic networks, trophic levels, interfirm credit

## Abstract

Understanding how credit flows through interfirm networks is critical for assessing financial stability and systemic risk. In this study, we introduce *DebtStreamness* (DS), a metric inspired by trophic levels in ecological food webs, to quantify the position of firms within credit chains. By viewing credit as the primary energy source of the economy, we measure how far credit travels through interfirm relationships before reaching its final borrowers. Applying this framework to Uruguay’s interfirm credit and financial institutions-firm networks, using credit registry and survey data from the Central Bank, we find that credit chains are generally short, with a tiered structure in which some firms act as intermediaries, lending to others further along the chain. We also find that local network motifs such as loops can substantially increase a firm’s DS, even when its direct borrowing from financial institutions remains the same. Comparing our results with standard economic classifications based on input–output linkages, we find that DS captures distinct financial structures not visible through production data. We further validate our approach using two maximum-entropy network reconstruction methods, demonstrating the robustness of DS in capturing systemic credit structures. These results suggest that DS offers a complementary ecological perspective on systemic credit risk and highlights the role of hidden financial intermediation in firm networks.

Significance Statement Credit flows through interfirm networks similarly to how energy circulates in ecological food webs between different species. Drawing on the concept of trophic levels, we introduce DebtStreamness (DS), a metric that captures the depth and complexity of credit chains. Unlike traditional production or interbank analyses, our approach uncovers hidden layers of financial intermediation and reveals how local structures, such as loops or extended chains, can amplify systemic vulnerabilities. Applying the metric to Uruguay’s credit network, we show that most credit chains are short but that specific motifs create concentrated fragilities. This ecological perspective provides a simple, robust tool for identifying critical firms and anticipating cascading disruptions, offering policymakers and researchers new insights into systemic risk and economic resilience.

## Introduction

In any economy, the production dynamics of goods and services depend on the complex relationships between firms. Often, the output of one firm is linked to the products or services of another. Every sector requires specific inputs to manufacture its goods, and then supplies those goods to other sectors to fulfill their specific needs (1). Input–output (IO) networks (2) illustrate how raw materials flow from primary sectors, such as agriculture and mining, to manufacturing sectors for processing, and finally to service sectors. This is akin to how nutrients flow through a food web, from primary producers (such as plants and phytoplankton) to various levels of apex consumers, such as herbivores and carnivores, sustaining the complex ecosystems observed in nature (3).

Both ecology and economics use network representations to capture the flow of resources, goods, or influence within such complex systems (4–6). To better understand the role of individual agents—whether species in an ecosystem or sectors in an economy—similar metrics have been developed to quantify their position within these networks.

In ecological food webs, species are connected by predator–prey relationships. A species’ position within this web is captured by its *trophic level*, indicating the relative position that it occupies in the ecosystem and their distance from primary energy sources, ie apex predators have a larger trophic level than phytoplankton (7–9).

In economics, DownStreamness and UpStreamness measure a sector’s position with respect to primary factors of production and final consumers, respectively (10). Specifically, UpStreamness measures the distance of a firm or sector from final demand: an upstream sector sells a small share of its output to final consumption and a large share to other sectors for producing intermediate output (11). Similarly, DownStreamness represents the distance of a given sector from the economy’s primary factors of production (12). These measures provide a clearer picture of how many intermediate stages a product or service passes through before reaching the final consumer, although the two measures have a complex and far from intuitive interplay (13).

In both ecological and economic networks, the structure of interactions and the position of nodes within the network are closely linked to the system’s stability and its response to exogenous shocks (14–16). May’s pioneering work on random ecosystems (17) highlighted how connectivity influences stability, providing a framework to discuss the observed robustness of real systems. For instance, Dunne et al. (18) showed that food web stability is influenced by the nonrandom distribution of interaction strengths, showing resilience when strong and weak links coexist. Similarly, Neutel et al. (19) found that food webs are organized such that long loops contain many weak links, which critically contributes to their stability. Allesina and Pascual (20) further stressed that a species’ position within the network, rather than its number of connections, is key to understanding its impact on co-extinctions.

In financial networks, similar questions about structure and stability have been extensively studied. (21) explored the Austrian interbank market and found power law dependencies in degree distributions with very low degrees of separation between nodes. References (22) and (23) documented core–periphery architectures in interbank networks, where core institutions are highly interconnected and peripheral nodes connect primarily to the core—a structure with important implications for systemic risk propagation.

In economics, Leontief’s foundational analysis showed that the response of an economy to a demand shock is determined by the structure of the IO network via the so-called Leontief inverse, which accounts for the paths through which the shock propagates between sectors (2). More recently, Acemoglu et al. (24) showed how aggregate macroeconomic fluctuations can emerge from such production networks, while Elliott et al. (25) argued that supply networks with intermediate productivity levels are particularly fragile at equilibrium. Building on this work, Acemoglu et al. (26) showed that network completeness can mitigate losses from smaller shocks through efficient use of excess liquidity, but that high connectivity no longer ensures stability when shocks are sufficiently large. Reference (27) further showed that integration and diversification have nonmonotonic effects on cascade extent at intermediate connectivity levels, with diversification initially enabling cascade transmission, but eventually providing insurance against failures. The literature on contagion and default cascades has grown substantially since the early work by Allen et al. (28) and Eisenberg et al. (29). Traditional cascading failure models such as those by Furfine (30) have been criticized for not capturing the propagation of distress before defaults occur (31, 32). To address this, Battiston et al. (14) introduced the DebtRank algorithm, which accounts for credit quality deterioration and was shown to better capture the buildup of systemic risk prior to the subprime crisis. Network simulation methodologies have also been developed for stress testing systems (32–34), allowing regulators to assess how shocks propagate through different network configurations.

The ecological perspective has recently gained traction in the study of complex economic and financial systems, where the concept of *trophic levels* is being adapted and enriched to understand structural stability and risk propagation. For example, Samson et al. (35) introduced the notion of trophic coherence—a measure of how neatly hierarchical a network is—as a key determinant of stability in economic and financial networks. These structural insights have practical implications for risk monitoring. Their work highlights that systems with higher trophic coherence tend to be more resilient to shocks, echoing patterns observed in ecological food webs. Beyond this, analyses of firm-level hierarchies and network topology have revealed that structural complexity itself can be a source of systemic risk, with certain configurations amplifying vulnerabilities within markets (36). These insights highlight the relevance of ecological frameworks in uncovering hidden fragilities in economic and financial networks.

Existing economic analyses largely focus on material flows in production networks or on direct financial exposures (interbank lending, derivatives) in financial networks, neglecting the equally critical pathways of trade credit flows between nonfinancial firms. Moreover, while ecological concepts have begun to inform the analysis of financial systems (35), a systematic framework to quantify positions within interfirm credit networks remains absent. In this study, we bridge this gap by introducing *DebtStreamness* (DS)—a metric inspired by trophic levels—to quantify a firm’s position within credit chains. Firms extend credit to each other based on various operational needs, and these credit relationships form a directed network of interfirm obligations. DS measures the distance separating a firm from financial institutions, which we consider the originator of credit in the system. When efficiently allocated, credit—acting as the primary “energy” source of the economy—flows through the network, fueling productivity and enabling economic activity. By analyzing DS, we gain a structured understanding of how credit flows from its primary source (banks and nonbank financial institutions) to the various firms that make up the economy. This approach not only captures the length of credit chains but also offers insights into potential bottlenecks and hidden financial intermediaries that could propagate or absorb exogenous and endogenous shocks.

In this context, we define a firm as a hidden financial intermediary when it borrows from financial institutions and, in turn, extends credit to other firms further along the credit chain, thereby transmitting bank funding through interfirm relationships that are not directly observable from standard balance-sheet or sectoral data. The “hidden” nature of this intermediation arises from two sources: (i) interfirm credit relationships are typically unreported or only partially disclosed and (ii) even when such data are available, standard analyses based on firm size, leverage, or sectoral classification do not reveal a firm’s position within the credit chain.

While DS is formally inspired by the concept of DownStreamness in IO economics (12), it substantially differs along several dimensions: (i) the object of analysis (financial credit networks rather than production networks), (ii) the reference node (financial institutions as primary credit originators rather than final demand or primary factors of production), and (iii) the level of granularity (firm-level rather than aggregate sectoral data), which together allow us to identify individual firms acting as hidden financial intermediaries, a dimension of systemic risk entirely invisible in production-based analyses.

We apply this framework to the interfirm credit network of Uruguay, leveraging a unique dataset collected by the Central Bank of Uruguay during a 2018 survey of the country’s 240 largest firms (37) and credit registry information. While the survey dataset is partial—each firm reports only its top three creditors—it nonetheless provides rare, granular insight into the structure of interfirm credit relationships. To assess the reliability of our results given this limitation, we complement our analysis with three maximum-entropy network reconstruction techniques, incorporating additional survey data on total interfirm credit exposure.

Our analysis reveals a tiered credit structure in Uruguay’s interfirm credit network: a group of firms borrows primarily from financial institutions and, in turn, provides credit to others. We also find a strong negative correlation between the share of a firm’s credit sourced directly from financial institutions and its DS. This finding is intuitive—greater reliance on credit from financial institutions leads to shorter credit chains. However, we also show that network-specific features can significantly affect a firm’s DS, highlighting the complex interplay between local structure and systemic credit flows. We also show that sector-level aggregation can obscure these patterns, emphasizing the importance of more granular data.

By comparing our results with a standard upstream and downstream classification of firm types based on production network analysis, we demonstrate that interfirm credit analysis provides complementary insights beyond what production-based approaches capture. Finally, we evaluate how exogenous shocks propagate through the interfirm credit network by simulating a contraction in financial institution credit. We find that shock propagation is heavily concentrated among firms with high DS, with loss rates escalating multiplicatively rather than proportionally to the initial shock. This threshold effect suggests that effective systemic risk monitoring should prioritize firms in the upper tail of the DS distribution, as they may represent the important sources of financial contagion.

By offering a fresh lens on credit flows, our work provides complementary insights to traditional production-based analyses and highlights firms whose network positions may render them critical to financial stability, regardless of their size. Our approach demonstrates that even partial network data can generate meaningful and policy-relevant systemic risk insights, illustrating the strength of network methods in extracting actionable information and reinforcing the value of investing in more systematic data collection infrastructure. This interdisciplinary framework opens new avenues for policymakers, regulators, and researchers aiming to monitor systemic risk in increasingly interconnected economies.

The article is organized as follows: Methods section introduces the definition of DS and describes the dataset used in our analysis. Results section presents our results at the level of the granular interfirm network, the sectoral level, and it includes robustness checks based on partially reconstructed networks. Finally, we conclude with a discussion of our main findings and their implications.

## Methods

In this section, we present the DS metric, describe the dataset, and outline the network reconstruction methods employed to address data incompleteness and validate the robustness of the methodology.

### DebtStreamness: measuring positioning in credit networks

To capture the flow of credit within an economy, we model the interfirm financial system as a *directed, weighted network* of *N* nodes, where each node represents a firm and each link denotes a credit relationship. Specifically, the network is encoded in a matrix *L*, where each element $L_{ij}$ reflects the amount borrowed by firm *i* from firm *j*. We further denote by $D_{i}$ the total amount of borrowing of firm *i*, and by $B_{i}$ the amount borrowed by firm *i* from financial institutions.

We focus exclusively on credit originating from *financial institutions (banks and nonbanks)* or *other firms* within the interfirm network, omitting alternative sources of credit such as bonds, private equity, or government loans. This assumption leads to the following accounting identity for each firm:


(1) $$D_{i}=B_{i}+\sum_{\;j=1}^{N}L_{ij}.$$

Our objective is to quantify a firm’s position within the credit network, interpreted as its average distance from financial institutions—the primary originator of credit, analogous to primary producers in ecological food webs. This “distance” reflects how many layers of interfirm credit a unit of currency traverses before reaching a given firm. Financial institutions serve as a natural reference point for measuring distance within the network.

To formalize this, we adapt the concept of DownStreamness—originally developed for IO production networks (11, 12)—to the context of interfirm credit flows.

Following Antras et al. (11), we therefore define the DebtStreamness ${\mathrm{DS}}_{i}$ of firm *i* as a weighted sum over all possible paths from financial institutions to firm *i*. Each path is weighted by its length (number of intermediaries) and by the fraction of credit transmitted along that path from financial institutions to firm *i*.

Let $\ell_{ij}=L_{ij}/D_{j}$ represent the fraction of firm *j*’s debt that flows to firm *i*. Then, the DS of firm *i* is given by:


(2) $$DS_{i}=\frac{B_{i}}{D_{i}}+2\;\sum_{j}\frac{\ell_{ij}B_{j}}{D_{i}}+3\;\sum_{\;jk}\frac{\ell_{ik}\ell_{kj}B_{j}}{D_{i}}+\ldots .$$

**Figure 1.**
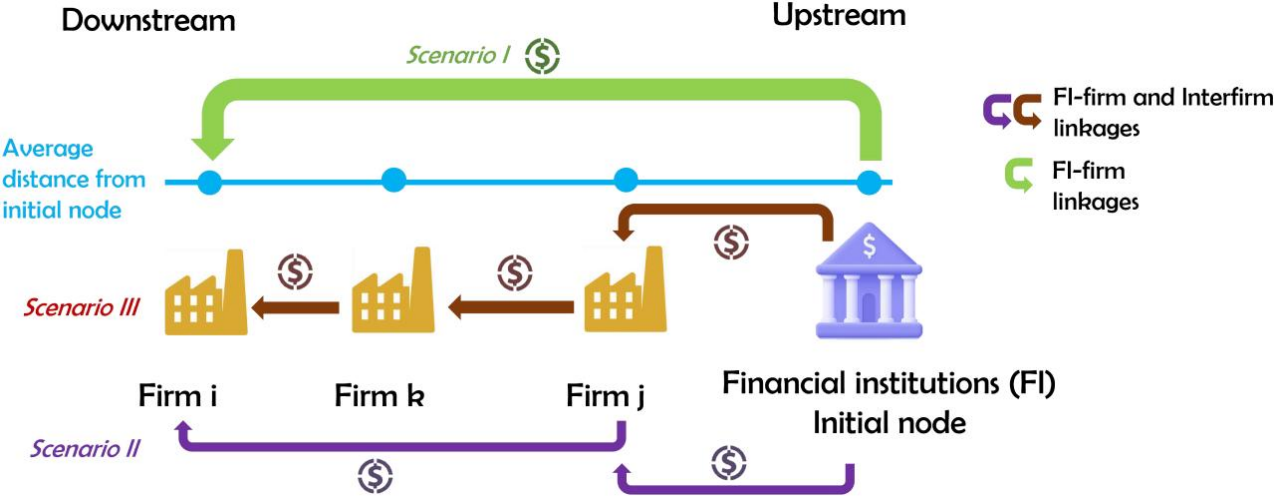
DS of firms. This diagram shows the gross debt value of firm *i*, positioned downstream in the final stage of the debt chain, relative to financial institutions, which are upstream and act as credit originators. We show three different toy scenarios from firm *i*’s perspective. Scenario I refers to the situation when firm *i* borrows from financial institutions (initial node). Scenario II reflects the scenario where *i* borrows from *j*, which in turn borrows from a financial institution. Finally, scenario III refers to the situation where *i* borrows from *k*, which borrows from *j*, which borrows from financial institutions (initial node).

On the right-hand side of the above equation, the first term accounts for the direct borrowing of firm *i* from financial institutions, the second term for the borrowing that is associated with credit chains or paths of length 2 (ie borrowing that is intermediated by one firm), the third term for paths of length 3 (intermediated by two firms), and so on. In Fig. 1, we provide a graphical illustration of the first three terms of Eq. 2, corresponding to different borrowing scenarios and length of credit chains.

Following (11), it can be shown (see Section S1) that the above definition is equivalent to the following expression:


(3) $${\mathrm{DS}}_{i}=1+\sum_{j}A_{ij}{\mathrm{DS}}_{j},$$

where $A_{ij}=L_{ij}/D_{i}$ denotes the share of firm *i*’s debt that belongs to firm *j*.

This mirrors the definition of *trophic levels* in ecology (13): just as a species’ trophic level equals one plus the average trophic level of the species it feeds on, the DS of a firm equals one plus the weighted DS of its creditors.

If we consider all nodes in the network i=1,…,N and collect the DS of all nodes in a vector $\vec{\mathrm{DS}}$, Eq. 3 can be written as


(4) $$\vec{\mathrm{DS}}=1+A\;\vec{\mathrm{DS}},$$

with solution


(5) $$\vec{\mathrm{DS}}={\left(1-A\right)}^{-1}\vec{1},$$

where 1 is the identity matrix and $\vec{1}$ a vector with all entries equal to one.

### Data

We apply this framework using a unique dataset from the *Economic Expectations Survey* conducted by the Central Bank of Uruguay in 2018 (37). The survey includes detailed credit relationship data for 240 large firms, each with over 50 employees. The survey excludes sectors such as primary industries, financial intermediaries, the public sector, and real estate. For each firm, the survey provides monthly information about:

- Total commercial debts and sales credit,
- Its *top three creditors and debtors*, along with corresponding amounts borrowed from and lent to them.

By incorporating both surveyed firms and their reported counterparties (ie top creditors and borrowers, many of which were not directly surveyed), we obtain an interfirm credit network of 1,072 firms. Although partial—as surveyed firms disclose only their top creditors and borrowers, with even less data for nonsurveyed firms—this dataset provides rare, granular insight into credit structures. Nonsurveyed firms appear in the credit network based on their reported relationships with the 240 surveyed firms: they are positioned upstream (downstream) when serving as creditors (debtors) to surveyed firms, or in both positions when maintaining different relationships with different surveyed firms. An important data limitation is that we do not observe direct credit relationships among the 832 nonsurveyed firms themselves, only their connections to the surveyed population. However, this limitation is substantially mitigated by the economic relevance of our sample: the 240 surveyed firms represent Uruguay’s largest enterprises, accounting for ∼60% of total economic activity and constituting the most systemically critical nodes in the interfirm credit network. Therefore, missing links among smaller nonsurveyed firms are unlikely to substantially affect the core network structure or our main findings. Moreover, a robustness check using network reconstruction techniques has been conducted, as shown later.

The data obtained through the survey are further complemented by the Central Bank Credit Registry database, which contains precise information about firms’ borrowing from financial institutions, both banks and nonbanks. Specifically, we have information regarding 11 banks and “other financial institutions”, including financial houses, credit management companies, and financial intermediation cooperatives. These institutions are incorporated into the credit network, allowing us to construct an enlarged network that represents the linkages between economic sectors and each financial institution, which we use to compute the borrowed amount $B_{i}$ for each firm *i*. Some firms in the dataset lack incoming paths from financial institutions. Consequently, their DS is undefined, and they do not contribute to the DS of other nodes in the system. These nodes have, therefore, been excluded from the analysis (see Fig. S1).

**Figure 2.**
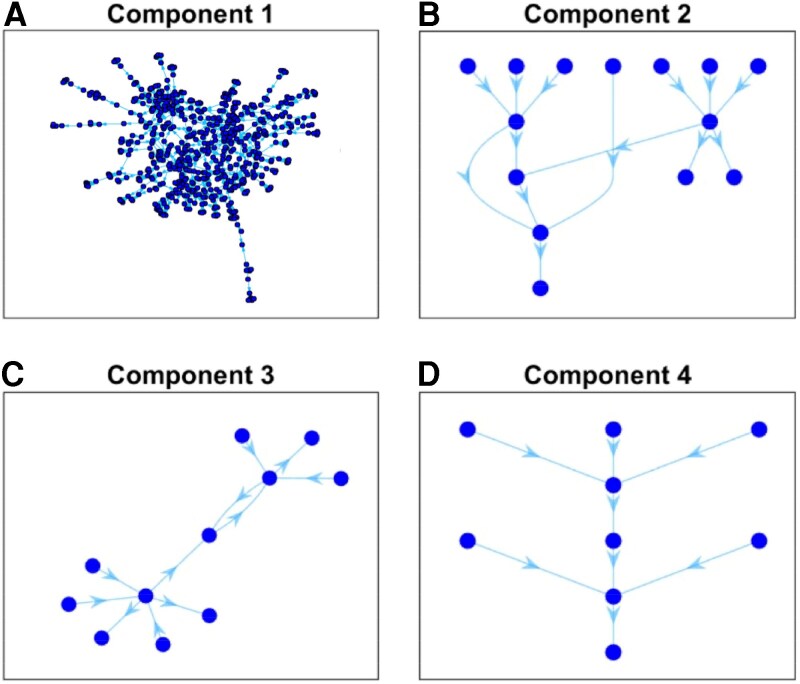
Network components. Main connected components of the interfirm credit network.

The resulting network comprises 843 nodes, and it is made of several components. In Fig. 2, we show four of these components, including the largest one (Fig. 2A) with 675 nodes.

**Figure 3.**
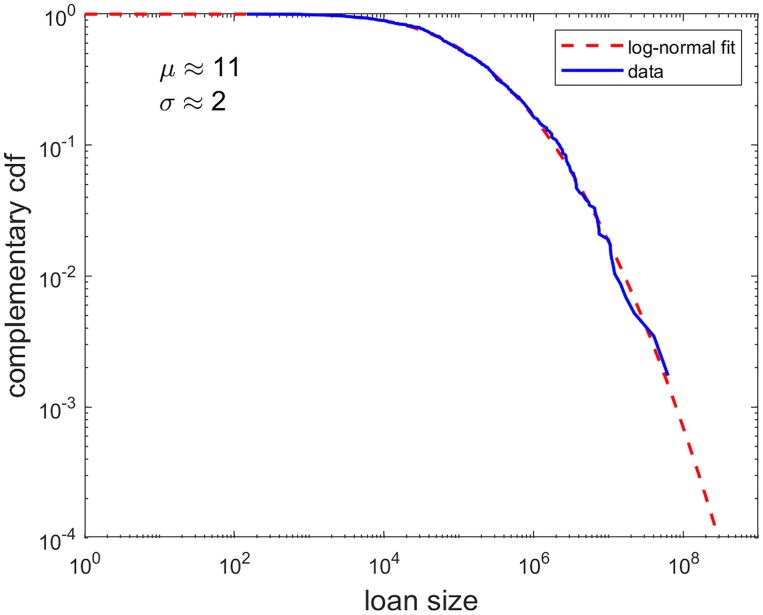
Distribution of interfirm credits. The data are well-fitted by a log-normal distribution of parameters μ≈11 and σ≈2.

The distribution of interfirm credits is well-fitted by a log-normal distributions of parameters μ≈11 and σ≈2 as shown in Fig. 3. The properties of our networks are compatible with typical observed behavior in interfirm networks (38).

**Figure 4.**
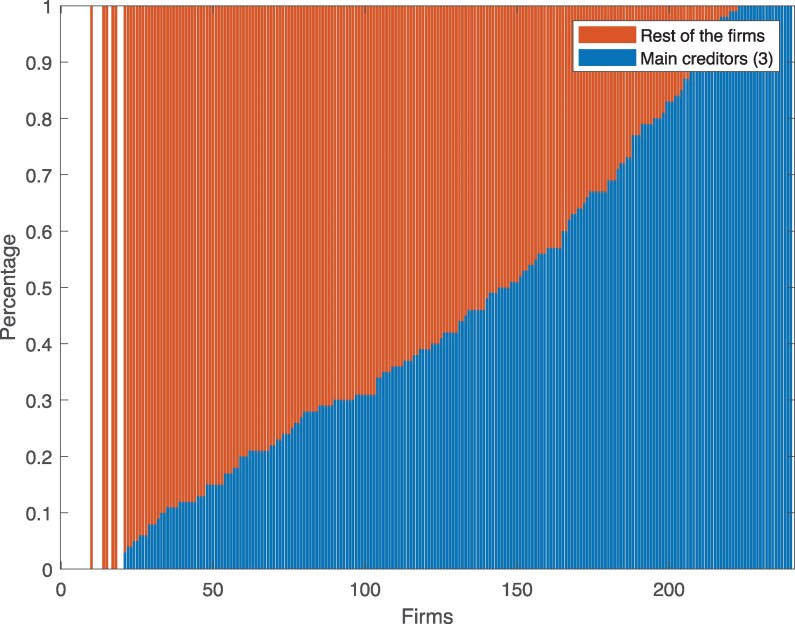
Interfirm credits by firm. Fraction of interfirm credit associated with the top three creditors of surveyed firms. The distribution is quite heterogeneous but on average the top three creditors of each firm account for about 50 of their interfirm credit.

In Fig. 4, we report the share of total interfirm credit accounted for by the top three creditors of each firm. While, on average, the top three creditors represent about 50 of a firm’s interfirm credit, the distribution is highly heterogeneous. This raises the question of how the residual credit—ie credit relationships beyond the top three—might affect our results. To address this, we conduct robustness checks on partially reconstructed networks that incorporate this additional information.

### Network reconstruction and robustness analysis

In financial network analysis, it is common to work with incomplete data, as full bilateral exposures between institutions are often unavailable due to privacy, reporting thresholds, or survey limitations (39, 40). To overcome this, network reconstruction methods—particularly those based on maximum entropy or sparsity assumptions—are widely employed to estimate the missing entries in the exposure matrix while preserving key known constraints (41).

In our case, the empirical dataset provides partial network information: for each surveyed firm, the top three creditors and debtors are disclosed. However, the total volume of interfirm credit for each firm is also reported. This allows us to test the robustness of our DS measure under different plausible reconstruction methods of the entire original credit network.

In particular, we denote by $L^{\mathrm{top}}$ the observed interfirm credit matrix, containing only the top three credit relationships for each surveyed firm. Let $F_{i}$ denote the total volume of interfirm credit borrowed by firm *i*, as reported in the survey. The residual, or unobserved, portion of credit for firm *i* is then given by:


(6) $$R_{i}=F_{i}-\sum_{j}L_{ij}^{\mathrm{top}}.$$

To explore how this residual credit may influence DS values, we construct two alternative partially reconstructed networks that represent two extreme scenarios of possible credit allocation.

#### Fully connected network reconstruction

In this case, we assume that the residual credit of each firm is uniformly distributed among all other firms not already listed as one of its top three creditors, hence filling uniformly all zero entries of the *i*th row of $L^{\mathrm{top}}$. Formally:


(7) $$L_{ij}^{\mathrm{full}}=\left\{ \begin{array}{cc} L_{ij}^{\mathrm{top}}, & \text{if}\;L_{ij}^{\mathrm{top}}>0\;,\\ \frac{F_{i}-\sum_{k}L_{ik}^{\mathrm{top}}}{N-3}=\frac{R_{i}}{N-3}, & \text{if}\;L_{ij}^{\mathrm{top}}=0, \end{array}\right.$$

where *N* is the total number of firms in the network.

#### Sparse network reconstruction

Here, we preserve the sparsity of the observed network. For each firm *i*, we determine the minimum number $N_{i}$ of additional creditors such that if $R_{i}$ is evenly distributed among them, each entry remains smaller than the smallest value in *i*’s top three observed credit links. This prevents the reconstructed links from dominating the observed structure. We then randomly assign the residual credit evenly across $N_{i}$ randomly selected zero entries in row *i* of $L^{\mathrm{top}}$.

#### Fully connected IO reconstruction

In this case, we use IO tables information for Uruguay to redistribute $R_{i}$ proportionally to the share of production reported in supply chain data.

These reconstruction methods allow us to test whether DS is sensitive to the unknown distribution of the remaining credit. In Results section, we compare the DS values computed from $L^{\mathrm{top}}$ with those obtained using the reconstructed networks. The resulting high correlations confirm that DS is robust to this class of uncertainty and supports the reliability of our findings, even under limited data availability.

Having established the DS framework and introduced the underlying data, we now turn to its empirical application. In the following section, we examine the structure of Uruguay’s interfirm credit network through the lens of DS—first at the firm level, then at the sectoral level—highlighting the emergence of hierarchical credit chains, identifying key intermediaries, and testing the robustness of our findings under alternative network reconstructions.

## Results

We now analyze the structure of the Uruguayan interfirm credit network through the lens of DS, the economic counterpart to trophic level. By examining both firm- and sector-level positions within the network, we identify patterns in credit flow hierarchies, assess the role of intermediaries, and evaluate how network features such as loops or aggregation affect systemic positioning and potential network fragilities.

**Figure 5.**
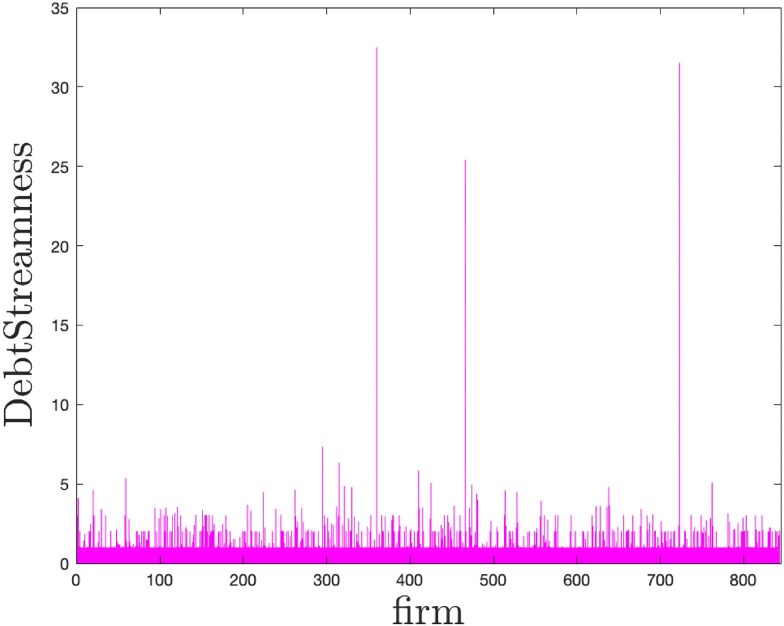
DS of firm network. DS of firms in the Uruguayan interfirm credit network. The average value of DS is 1.67, with three outliers with DS>20.

In Fig. 5, we show the DS of firms within the Uruguayan interfirm credit network. The average DS is 1.67, indicating that, on average, firms are relatively close to the primary credit source—financial institutions. The minimum value of DS is 1, which corresponds to a firm that borrows from financial institutions only, and most firms have DS values below 6, suggesting rather short credit chains. However, we identify three outliers with DS above 20, reflecting their remote, multilayered positioning within the credit web. These firms are further away from financial institutions along a more complex credit chain.

To further characterize the firms in our sample, we examine how DS relates to firm-level attributes and standard network measures (see Supplementary information). In particular, we compare DS across credit risk categories and find that firms with higher credit risk tend to occupy more peripheral positions in the credit network, consistent with their greater reliance on indirect borrowing chains. We also compare DS with several standard centrality measures, including degree, strength, eigenvector, betweenness, closeness, PageRank, and a simplified DebtRank. While DS is positively correlated with degree and strength, its correlations with other centrality measures are more moderate, indicating that it captures a distinct structural dimension of firms’ positions within the credit network. Details of these analyses are reported in Figs. S3 and S4.

As mentioned earlier (see Fig. 2 in Methods section), the interfirm credit network is fragmented into several disconnected components, each representing a self-contained subsystem of interfirm credit relationships. Within a given component, the DS of firms depends solely on the internal structure, since no credit paths link them to firms outside the component, allowing credit transfers from one component to another. This structural isolation resembles ecological subnetworks or isolated food chains, where energy flows are confined within distinct ecosystems.

This separation allows us to analyze each component independently, shedding light on how local network topology influences firms’ positions along credit chains. In what follows, we first focus on the largest component, which captures the majority of firms and illustrates the typical hierarchical patterns of DS across the economy. We, then, turn to a smaller component that contains the three most extreme outliers—firms positioned farthest from financial institutions.

By contrasting these two cases—a large, relatively hierarchical component and a smaller, structurally atypical component—we highlight how fine-grained network features, such as loops, can amplify credit chain length and affect systemic risk. This comparative approach highlights the importance of granular, component-level analysis in uncovering hidden vulnerabilities that would otherwise be masked in aggregate statistics.

### Largest component

**Figure 6.**
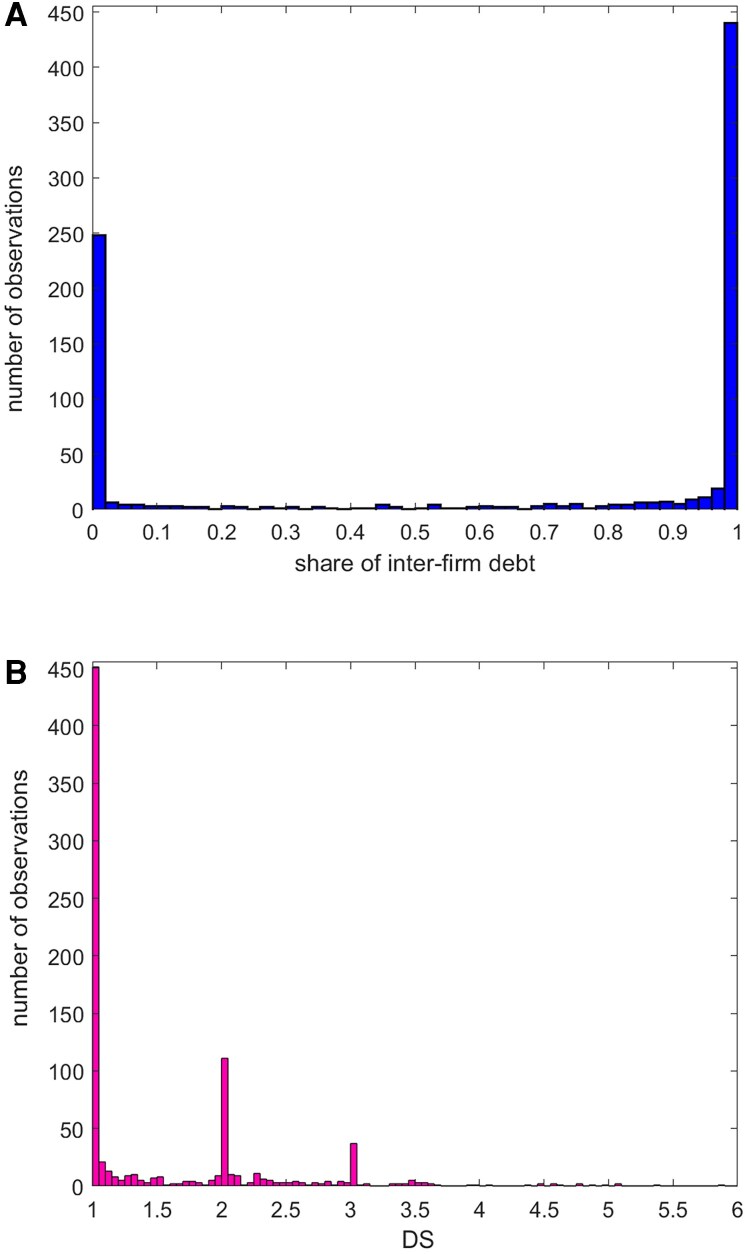
DS of firms in largest component. A) Histogram of the share of interfirm credit for firms in the largest component. Approximately 55 of firms primarily rely either on interfirm credit or direct borrowing from financial institutions, while the rest use a mix of both. B) Histogram of DS values for firms in the largest component. The three peaks at DS=1,DS=2,DS=3 suggest a hierarchical structure in the credit chains.

The top panel of Fig. 6 shows the histogram of the share of interfirm debt for nodes in the largest component. The distribution is bimodal: ∼25 of firms borrow very little from other firms, while around 30 heavily rely on interfirm debt. The remaining 45 firms are spread between the two extremes. These two financing sources, interfirm credit and credit from financial institutions (banks and nonbank financial institutions) mostly exhibit a substitute relationship, which is in accordance with the results found in Refs. (42) and (43). The likelihood of a firm obtaining credit from financial institutions may depend on its assets available to secure a loan. In cases where a firm lacks sufficient assets or during an economic downturn, it may become difficult for the firm to secure financing from financial institutions (44). Instead, firms may resort to interfirm credit, suggesting that it acts as a substitute for credit from financial institutions when a firm cannot obtain a loan. Without alternative funding sources such as financial institutions or investors, firms become more dependent on interfirm credit to continue their operations (45). In Ref. (42), the authors find that in emerging markets and developing economies in Asia, interfirm credit is an important source of financing and serves as a substitute for credit from financial institutions.

The distribution of DS values is shown in the bottom panel of Fig. 6. The peaks around 1, 2, and 3 suggest a layered, hierarchical structure in the flow of credit from financial institutions to firms. The highest peak, around 1, confirms that most firms primarily borrow directly from financial institutions. The peak near 2 corresponds to firms that, in turn, borrow significantly from firms with a DS of ∼1. Similarly, the peak around 3 represents firms borrowing from those with a DS of about 2.

**Figure 7.**
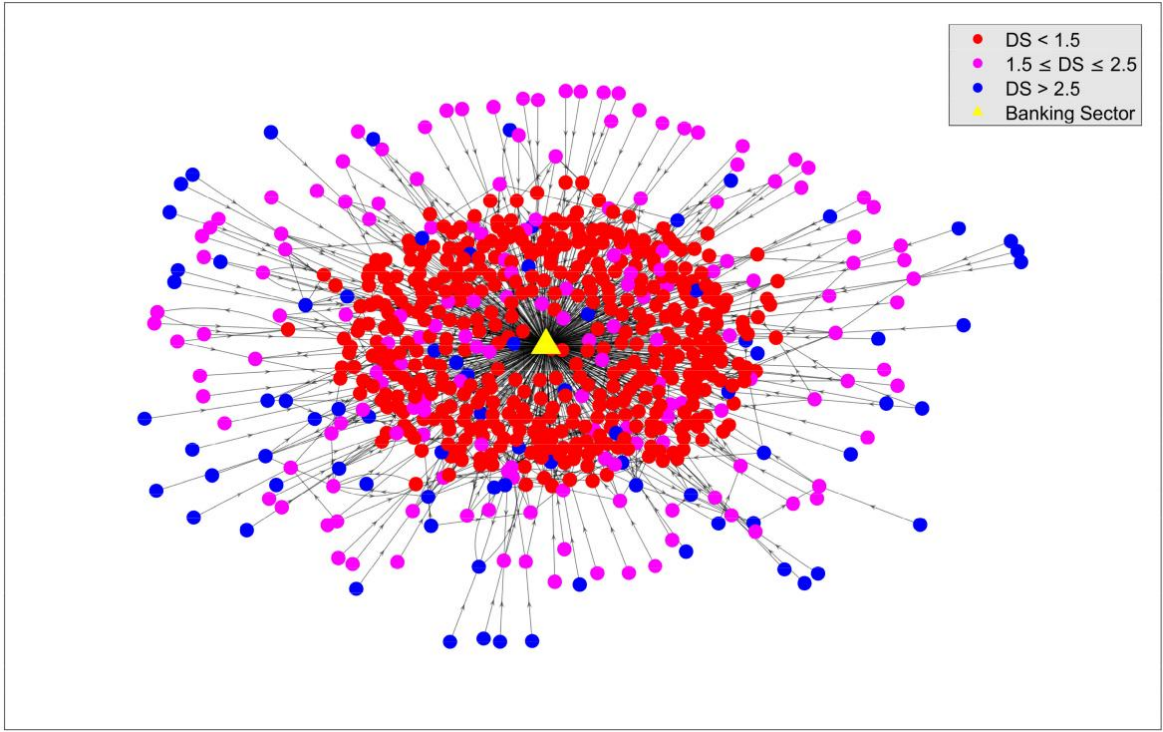
Visualization of the interfirm credit network. Largest component of the interfirm credit network. Nodes represent firms grouped according to DS ranges: DS<1.5, 1.5≤DS≤2.5, and DS>2.5. Financial institutions (shown as a triangle) serve as primary credit originator.

This stratified structure is also visually apparent in Fig. 7, where nodes are color-coded by DS value. Firms closer to financial institutions (yellow triangle) occupy central, red-colored positions, and have DS<1.5. Firms relying on extended intermediation form the outer layers and populate the peripheral network regions in blue and purple showing respectively a DS 1.5≤DS≤2.5 and DS>2.5.

The structure highlights a tiered flow of credit, with firms closer to financial institutions exhibiting lower DS values, while peripheral firms are associated with higher DS values due to longer credit chains.

### Component with outliers

Having analyzed the largest component, where DS values exhibit a clear, layered hierarchy, we now turn to a smaller but particularly interesting subnetwork—one that contains the firms with the highest DS values in the entire dataset (see Fig. 5). Studying this component offers a complementary perspective: it allows us to see how localized structural anomalies, such as extended chains and feedback loops, can dramatically alter a firm’s systemic positioning, similarly to how isolated clusters or strong cycles in ecological networks may distort energy flows and destabilize local food webs (46).

**Figure 8.**
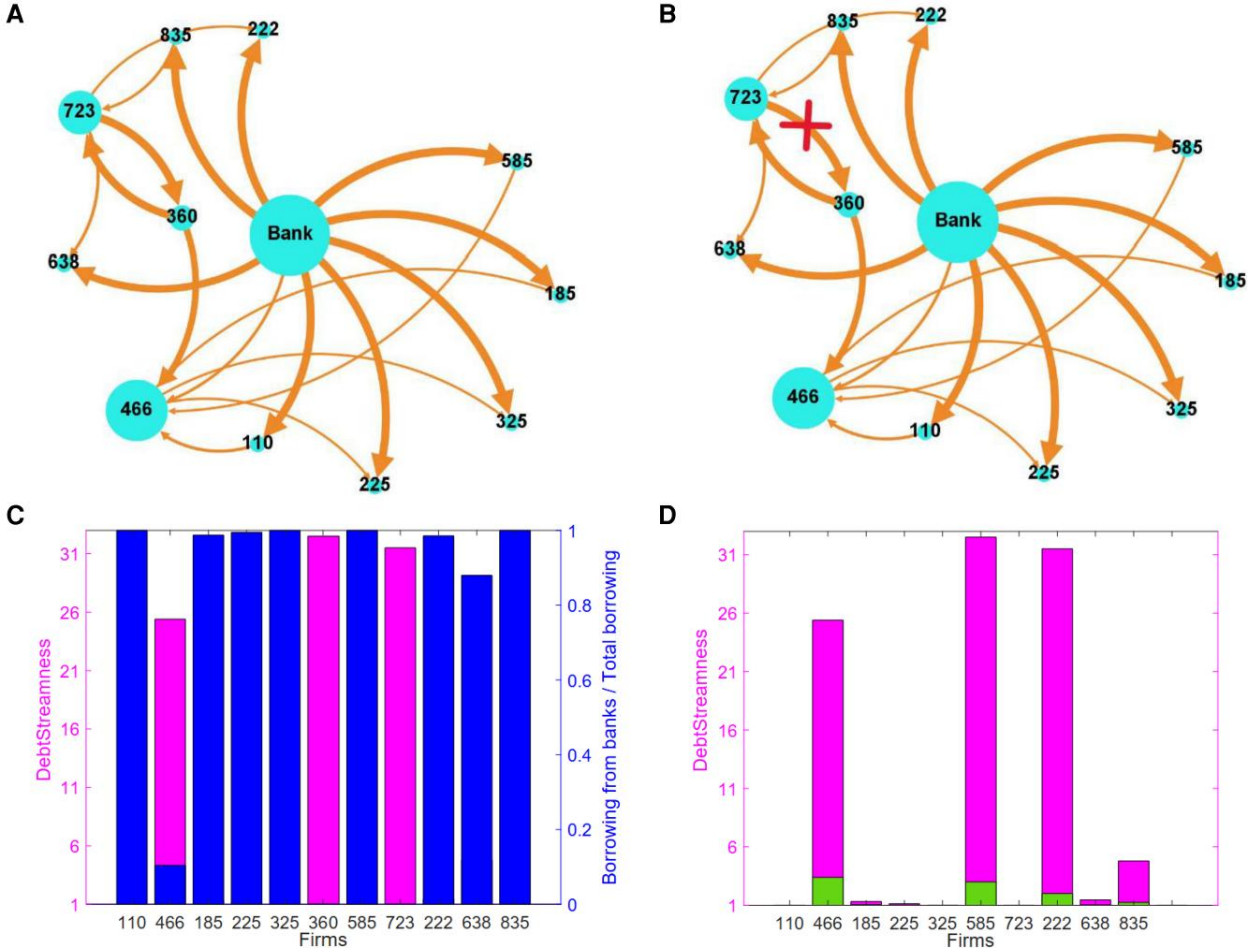
Loop effect on DS. A) Network representation of the component with the three outliers. A node’s size represents its total borrowing, and links indicate lender–borrower relationships. Two of the outliers do not borrow directly from financial institutions and form a loop in the network. The third outlier primarily borrows from one of the other two. B) Graph representation of the component with the three outliers after removing the loop between two of them. C) Bars associated with the right *y*-axis (blue) show the fraction of a firm’s total borrowing that comes directly from financial institutions, while bars associated with the left *y*-axis (pink) represent the firm’s DS value. The three outliers primarily rely on interfirm credit. D) Comparison of DS values in the original component (pink bars) versus the modified component with the loop removed (lower green bar). Removing the loop significantly reduces the length of credit chains.

The top left panel of Fig. 8 shows the component with the three outliers and illustrates their connections in the network. The direction of arrows in the figure goes from the lender to the borrower, and a representative node of the banking system is included at the center. We see that two of the outliers (nodes 360 and 723) are not directly connected to financial institutions, while the third (node 466) mostly borrows from node 360. Additionally, the two outliers with no direct borrowing from financial institutions are involved in a loop of length two, reinforcing credit circulation between them and increasing their DS. Such loops allow credit to circulate repeatedly among firms before reaching downstream nodes, effectively amplifying their DS (see Supplementary information for a simple illustration). Two main factors contribute to their increased DS: (i) paths from financial institutions to these three firms are longer due to intermediaries and (ii) the sum in Eq. 3 will contain infinite terms, corresponding to paths that repeatedly go through the loop.

In the remaining panels of Fig. 8, we explore these two aspects separately. In the bottom left panel of Fig. 8, we plot for each firm in the component its DS (pink bars) and the fraction of its debt borrowed directly from financial institutions (blue bars). We observe a strong negative correlation between the two quantities, which is around −0.99. This seems to suggest that, for the system at hand, the network structure is not that important; rather, in agreement with the results of Bartolucci et al. (10, 47, 48) obtained for related centrality measures, knowing the fraction of money that a firm directly borrows from financial institutions is enough to estimate, on average, its DS with good approximation. However, we will now see that certain details of the network can make a significant difference in terms of the DS of specific nodes. To this end, we compute the change in DS of nodes in the component that would occur if we removed one loop by removing the link from node 723 to node 360. This is shown in the right panels of Fig. 8. By removing the loop, we observe a significant reduction in DS, with an average DS of the component that decreases from 9.28 to 1.52. The presence of loops can dramatically shift a firm’s position within the credit hierarchy: this result highlights the disproportionate impact of small topological motifs on systemic positioning, echoing findings from ecological stability studies where feedback loops are known to critically influence the robustness and resilience of food webs (19). From a financial risk perspective, loops represent operational and liquidity risk rather than pure credit risk, since gross exposures remain relevant even when net credit positions are small (28, 29). Timing and liquidity mismatches can generate rollover risk, and cash-flow constraints may arise when expected payments are delayed, causing operational disruptions even under zero net positions. Moreover, loops involving many firms may act as contagion amplifiers, allowing shocks to circulate repeatedly through the network (14, 49), and signal hidden financial intermediation of systemic importance.

In summary, while DS and the share of borrowing from financial institutions are strongly correlated on average, the network structure can nonetheless have a substantial impact on the DS of individual firms. In this section, we focused on a small component of the network to illustrate the mechanism. However, a similar pattern holds more generally. For example, in the largest component of the network, the Pearson correlation between DS and the share of borrowing from financial institutions is around −0.8, indicating that DS reflects network structure beyond the size of direct borrowing from financial institutions.

### Sector analysis

While firm-level analysis reveals a fine-grained credit hierarchy, aggregating firms by sector allows us to explore whether such structure persists at higher organizational levels. To perform sectoral analysis, starting from the matrix $L_{ij}$ of interfirm credit, we construct the aggregate matrix $L^{\left(s\right)}$, where $L_{kt}^{\left(s\right)}=\sum_{i\in k}\sum_{\;j\in t}L_{ij}$ is the aggregate amount borrowed by firms of sector *k* from firms of sector *t*, and we define the total debt of sector *k* as $D_{k}^{\left(s\right)}=\sum_{i\in k}D_{i}$. After this aggregation, we can proceed as before to define the DebtStreamness ${\mathrm{DS}}_{k}^{\left(s\right)}$ of sector *k* as


(8) $${\mathrm{DS}}_{k}^{\left(s\right)}=1+\sum_{t}A_{kt}^{\left(s\right)}{\mathrm{DS}}_{t}^{\left(s\right)},$$

where $A_{kt}^{\left(s\right)}=L_{kt}^{\left(s\right)}/D_{k}^{\left(s\right)}$.

**Figure 9.**
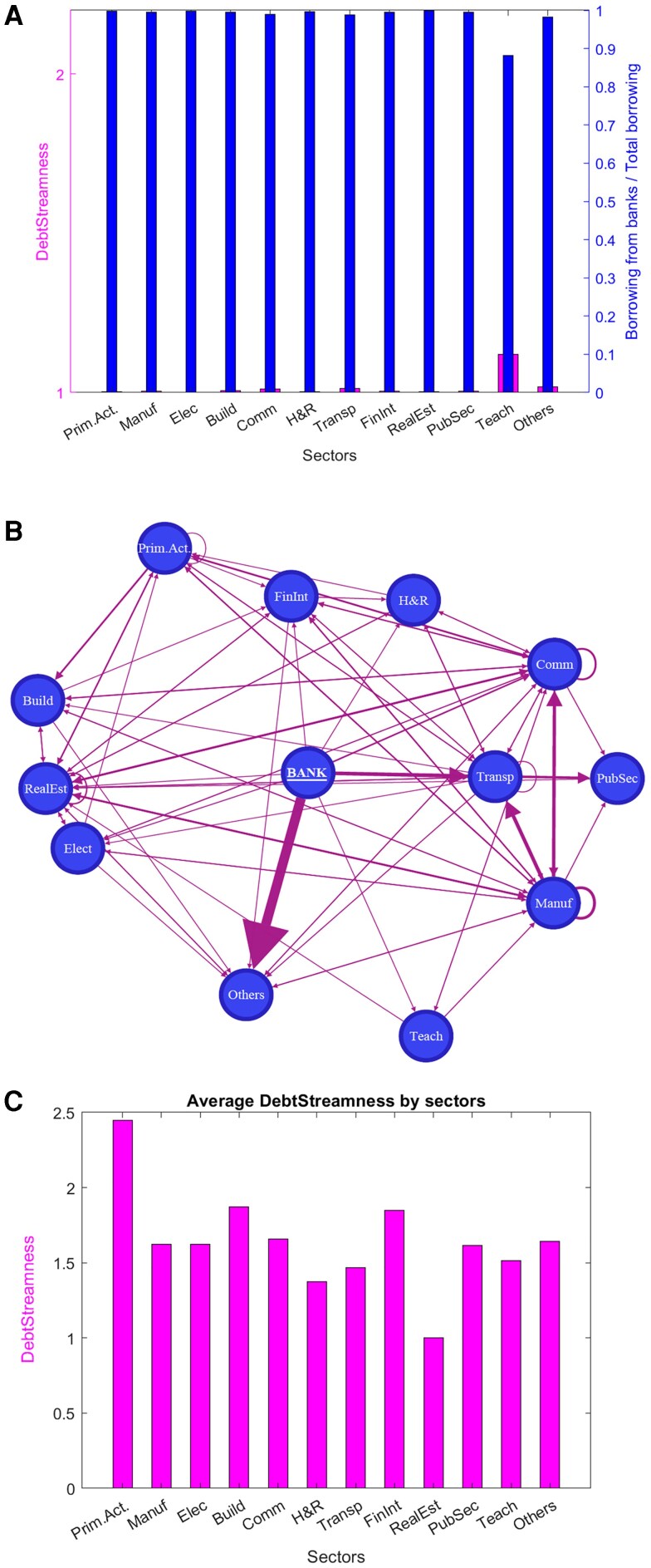
Sector aggregation. A) DS and bank-debt ratio of sectors. B) Network component visualization aggregated by sector with the bank node as originator. C) Average DS by sector.

The list of sectors in our dataset, number of firms, and the amounts borrowed by each sector from financial institutions and firms is reported in Table 1,^a^ and the results of this aggregation are shown in Fig. 9.

**Table 1.** Sectoral aggregate firm credit information.

Sector	Nodes	Ratio of firms’ borrowing from FIs to its total borrowing (%)
Primary activities	64	18.9
Manufacturing	267	31.7
Electricity	9	3.4
Building	32	8.2
Commerce	297	15.6
Hotels and restaurants	17	1.3
Transportation	104	8.3
Financial intermediation	7	0.2
Real estate	2	1.2
Public sector	29	5.4
Teaching	15	0.0
Others	229	5.7
Total	1,072	100.0

Ratio of firms’ borrowing from financial institutions (FIs) to its total borrowing and number of firms per sector.

In the upper panel of Fig. 9, we show DS alongside the share of borrowing from financial institutions for each sector. In the middle panel, we have the corresponding sectoral-level network representation with financial institutions node as credit originator. The figure shows that most sectoral borrowing comes from financial institutions rather than interfirm relationships, resulting in DS values close to 1 across all sectors. This pattern is also evident in the middle panel, where all sectors are directly connected to the node representing the banking system. The trivial credit chain structure at the sectoral level suggests that key interfirm credit relationships are obscured in aggregated data, highlighting the importance of fine-grained data to accurately capture the underlying economic dynamics.

The bottom panel of Fig. 9 shows the average DS by sectors. We found a meaningful cross-sectional variation in DS emerges across industries. Primary Activities, Construction (Building), Communications, and Financial Intermediaries display notably higher average DS values, indicating that firms in these sectors tend to occupy more peripheral positions from the banking sector in the credit network and are therefore more reliant on multistep intermediation chains. Sectors with higher DS exhibit greater dependence on trade credit and interfirm channels. This pattern aligns with theoretical expectations, as trade-oriented and input-intensive sectors such as construction and primary activities (50–52) tend to be more deeply embedded in credit intermediation networks, relying more heavily on supplier and buyer credit chains than on direct financing from financial institutions (53–55).

By contrast, sectors such as Real Estate (reflecting its direct reliance on bank mortgage and project financing), H&R, and Transport show lower average DS values, suggesting their interfirm credit relationships remain relatively close to the banking source. Although the cross-sectional variation is not extreme, it is economically meaningful and corroborates the view that industry structure plays a significant role in shaping firms’ positions within the credit network.

While our DS analysis captures how far sectors are positioned from the primary source of credit (banks and nonbank financial institutions), it is also insightful to compare this financial hierarchy with more traditional classifications of economic structure. In particular, sectoral roles within production networks—whether primarily input suppliers (upstream), final good producers (downstream), or key intermediaries—may influence their borrowing patterns and exposure along credit chains.

We adopted the classification of sectors based on the Uruguayan economic structure analysis from the Central Bank of Uruguay (56), and we grouped the sectors into three categories: Upstream, Key sectors, and Downstream. We labeled as “Others” the nodes for which sector classification is unknown. In this classification, sectors are categorized using the Rasmussen methodology (57) or the Backward (BL) and Forward Linkages (FL) approach. The BL measures how much a sector depends on its suppliers, while the FL measures how much a sector supplies inputs to other sectors. If BL>1, it indicates that sector *j* has a stronger backward linkage than the economy’s average, meaning it is heavily dependent on other sectors for inputs. Similarly, if FL>1, it indicates that sector *i* has a stronger forward linkage than the economy’s average, implying that many downstream firms depend on it. Based on these linkages, if BL>1 and FL<1, the sector is classified as upstream, as it provides inputs to other sectors but do not have strong downstream dependencies. If BL<1 and FL>1, the sector is classified as downstream, as it has a strong dependence on upstream sectors but contributes less to their input demands. Finally, if BL>1 and FL>1 , the sector is considered a key sector, meaning it plays a crucial role in both directions, serving as a both major supplier and consumer in the economy. This structural classification provides an independent perspective on economic positioning, allowing us to assess whether a sector’s DS aligns with its traditional production role, or reveals hidden vulnerabilities not visible through production linkages alone.

Here, we adopt the production network as a benchmark give the wide availability of the data and their importance in characterizing supply chain and economic sectors’ importance in an economy. Although one might a priori expect production and credit linkages to be closely aligned, using IO data as a proxy for commercial credit interlinkages can be misleading: the production network is generally much denser than the commercial credit network and is likely to overestimate indebtedness linkages, since the use of inputs from another sector does not necessarily imply financing through commercial credit (58).

**Figure 10.**
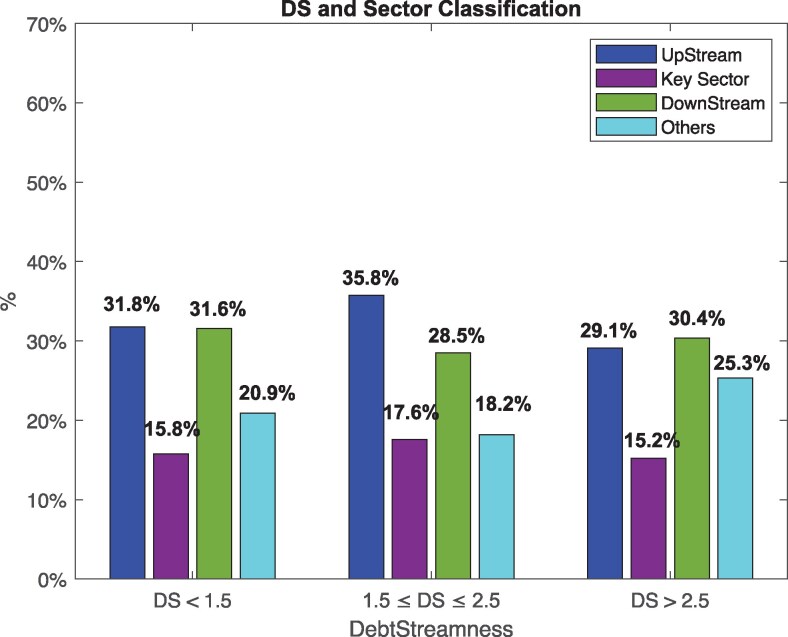
Sector classification by DS. This plot represents the sector classification of firms and their DS, categorized into UpStream, Key sector, DownStream, and Others with no classification available across three DS ranges: DS<1.5, 1.5≤DS≤2.5, and DS>2.5.

Figure 10 shows how sector classifications based on production linkages map to firms’ positions within the credit hierarchy, as captured by DS. Across the three DS ranges (DS<1.5, 1.5≤DS≤2.5, and DS>2.5), upstream and downstream sectors appear in roughly equal proportions. However, the middle DS range (1.5≤DS≤2.5) contains a noticeably higher share of upstream sectors, suggesting that these firms often combine both bank loans and interfirm credit. When DS>2.5, the downstream sector is slightly more likely to obtain financing from other firms and is further from the original credit source.

This result suggests that upstream sectors may enjoy more diversified or proximate access to credit, while downstream sectors are relatively more dependent on cascading interfirm lending—farther from the original source of credit. This finding reinforces the value of DS as a complementary lens to production-based classifications, revealing differences in financial positioning not captured by IO linkages alone.

### Robustness analysis: partially reconstructed networks

So far, our analysis has been based on the network constructed by linking each surveyed firm to its top three creditors and borrowers. However, as described in Methods section, we have also access to survey data regarding information on each firm’s *total volume* of interfirm credit, allowing us to estimate the extent of missing links in the observed network.

Following the reconstruction procedures introduced in Methods section, we conduct robustness checks by constructing three alternative partially reconstructed networks:

1. A fully connected reconstruction, where each firm’s residual credit is spread uniformly across all non-top-three partners.
2. A sparse reconstruction, where residual credit is assigned to a minimal number of partners, maintaining consistency with observed credit intensities.
3. A fully connected reconstruction by using IO tables data to systematically impute missing credit relationships based on sectoral production patterns.

These reconstructed networks allow us to test whether the structure of missing credit relationships could effectively distort the systemic positioning of firms as measured by DS.

**Figure 11.**
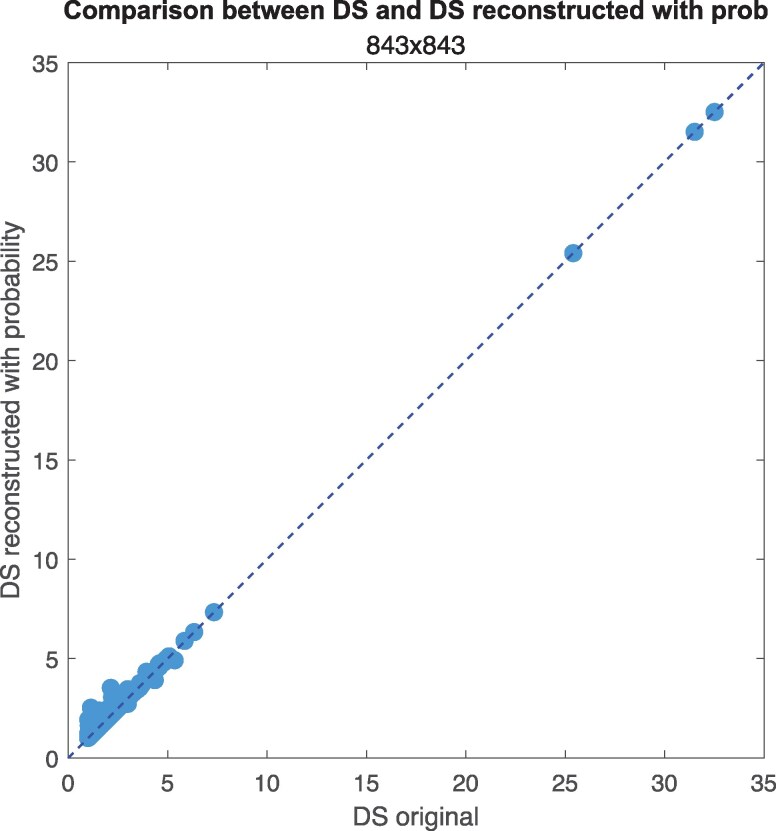
DS in reconstructed networks. Comparison of DS computed from the network with only top three creditors and the partially reconstructed networks. Here, we compare the original network with a partially reconstructed fully connected network.

The results, reported in Fig. 11 (fully connected) and in Fig. S5 (sparse and IO reconstruction), show the original DS values (computed from the observed top-three debtors/creditors network, $L^{\mathrm{top}}$) against those obtained from the three reconstructed networks. In the three cases, we observe extremely high correlations: Spearman correlations are 0.99 for both full and sparse networks and 0.98 for the IO network, and Kendall correlations are equal to 0.95, 0.96, and 0.95 for full, sparse, and IO network, respectively. The residual represents numerous smaller credit relationships that are less significant in determining firms’ positions within the credit hierarchy (see Fig. S2, where we compare the total with the residual credit, and we show the distribution of the latter).

This high degree of robustness is consistent with two key observations: (i) top creditors already account for a substantial share of interfirm credit, and (ii) as shown earlier, firms are generally located close to financial institutions in the credit hierarchy, limiting the potential for long, complex chains to form through the missing links.

Overall, the strength of the agreement across reconstructed networks confirms that our findings on DS—and the associated insights into credit chain structure and systemic positioning—are not sensitive to the partial nature of the available data. This reinforces the practical value of the DS metric for economic network analysis even in settings with incomplete information, a common feature in real-world data collection.

### Commercial credit stress-test results

To quantify how an exogenous shock propagates through the interfirm credit network and how losses depend on firms’ DS, we implement a stress-test analysis. Specifically, we model an exogenous contraction in credit supplied by financial institutions and track how losses propagate iteratively through the network according to the following DebtRank-like dynamics (14, 59):


(9) $$h_{i}^{\left(t\right)}=\min\left(1,s\;\frac{B_{i}}{D_{i}}+\alpha \sum_{j}\Lambda_{ij}\;h_{j}^{\left(t-1\right)}\right),$$

where $\Lambda_{ij}=W_{ij}/D_{i}$ is the share of firm *i*’s debt owed to firm *j*, $h_{i}$ denotes the fractional credit loss of firm *i*, *α* denotes the sensitivity of firms’ trade credit supply to upstream credit losses (ie how much firms reduce trade credit when their own credit is cut), *s* is the size of the credit contraction originating from financial institutions, $B_{i}$ is the amount borrowed by firm *i* directly from financial institutions, and $D_{i}$ is the total debt of firm *i*. In the simulations reported below we set α=1.15, and s=0.10, corresponding to a 10 contraction in credit supplied by financial institutions.

**Figure 12.**
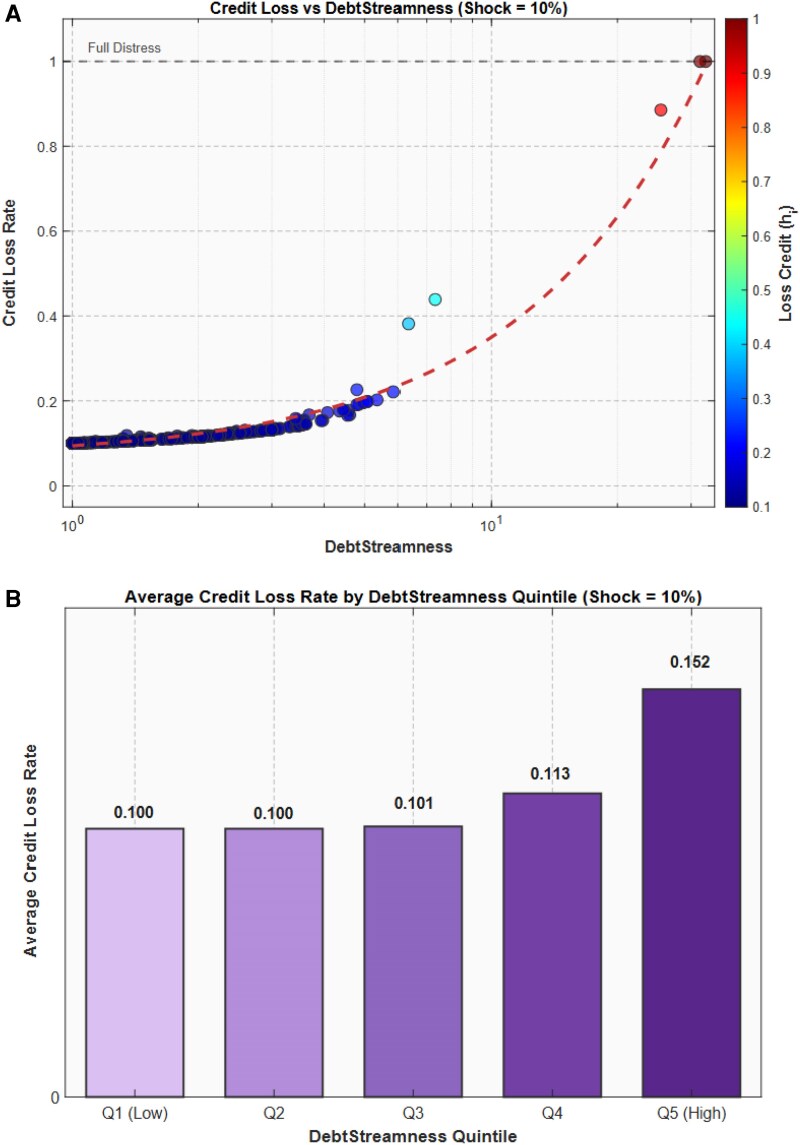
Credit losses and shock propagation. A) Credit loss rate versus DS under a 10% credit shock. The horizontal axis is displayed on a logarithmic scale. The dashed red curve represents a linear fit between credit loss rate and DS. Firms with higher DS tend to experience larger credit losses, while most observations remain concentrated at low loss levels and a small number of high-DS firms approach the upper boundary of distress. B) Average credit loss rate by DS quintile under a 10% credit shock. The first three quintiles display very similar loss rates close to the initial shock size, whereas firms in the highest quintiles experience larger losses, consistent with shocks accumulating along longer credit chains as they propagate through the network.

Figure 12A shows the relationship between DS and the credit loss rate under the 10 credit shock. The horizontal axis is displayed on a logarithmic scale. The dashed red line represents a linear fit between credit loss rate and DS. Firms with higher DS tend to experience larger credit losses following the shock, while most observations remain concentrated at low loss levels. A small number of high-DS firms approach the upper boundary of distress. This pattern is consistent with shocks propagating along credit chains. When firms respond to credit shortfalls by reducing their lending more than proportionally to the loss they experience (α>1), the propagation becomes amplified along the chain. By contrast, when α<1, propagation is dampened and the direct exposure to bank credit becomes more important, so that the relationship between DS and losses would tend to reverse.

Figure 12B reports the average credit loss rate across five DS quintiles under the same 10 credit shock. The first three quintiles display very similar loss rates close to the initial shock size, whereas firms in the highest quintiles experience larger losses.

These stress-test results show that credit shock propagation is heterogeneous across the network and strongly associated with firms’ DS. Firms with higher DS values experience larger cumulative losses following the initial credit contraction, reflecting the fact that shocks compound along longer credit chains as they propagate through the network. This has implications for systemic risk monitoring: a policy or regulatory framework that focuses exclusively on firm-level balance sheet size may overlook the role that network position plays in determining firms’ exposure to credit shocks.

## Conclusions

Drawing inspiration from ecology, this work develops an interdisciplinary approach to studying financial networks by adapting concepts from food web analysis—specifically, the idea of trophic levels—to the flow of credit between firms. In ecological systems, trophic levels capture how energy cascades from primary producers through successive layers of consumers; similarly, we conceptualize financial systems as structures in which credit originates from financial institutions (banks and nonbanks) and flows through firms via interfirm lending relationships.

Building on this analogy, we introduced the metric of DS to quantify the hierarchical structure of credit chains within an economy. DS measures a firm’s average distance from financial institutions—the origin of financial “energy”—across all possible credit paths in the interfirm network. Our measure provides a tool for mapping financial dependencies and systemic positioning in a manner that complements traditional production network analyses.

As a use case, we applied our metric to the analysis of interfirm credit in Uruguay, using data from the 2018 Economic Expectations Survey conducted by the Central Bank of Uruguay. Our analysis uncovers the following three key findings. First, credit chains are generally short: the average DS is close to 1.7, indicating that most firms rely predominantly on direct bank financing. However, we also observe a tiered structure, where some firms borrow primarily from financial institutions and, in turn, provide credit to others. Second, local network motifs—notably feedback loops—can inflate DS dramatically for specific firms, creating hidden pockets of increased systemic exposure despite similar levels of direct bank borrowing. Third, in spite of substantial missing data on smaller credit relationships, DS remains highly robust under alternative network reconstructions, demonstrating its practical applicability when only partial information is available. Our stress-test analysis further shows that DS is a useful tool for identifying systemic vulnerability. Effective risk monitoring should therefore prioritize the surveillance of firms in the upper tail of the DS distribution, as these firms may represent potential sources of financial contagion within the network. When we simulate a contraction in financial institution credit, the propagation of losses is heavily concentrated among high-DS firms, with losses increasing multiplicatively with DS rather than proportionally to the initial shock. This pattern suggests that dense interconnections of high-DS nodes in the credit network actively propagate credit distress across the network.

Moreover, DS offers insights that go beyond conventional production-based classifications of firms as upstream or downstream. These findings could support financial regulators and policymakers in identifying firms that act as hidden intermediaries in the credit network—entities that may not appear systemically important based on size alone, but whose network position makes them critical in the transmission of financial stress.

Although recent events such as the COVID-19 pandemic and the global energy crisis have drawn renewed attention to the fragility of supply chains and economic interdependencies, our findings show that financial flows—and specifically credit relationships—constitute an additional layer of vulnerability and resilience that deserves closer attention.

Our analysis represents an initial step toward a more comprehensive understanding of interfirm credit relationships. While we acknowledge that more comprehensive longitudinal data would enable richer dynamic analysis, the scarcity of such data in most contexts makes our findings particularly relevant: we demonstrate that meaningful insights about systemic vulnerabilities can be extracted from partial network information available to policymakers today. In this context, our contribution is thus twofold: establishing the analytical feasibility and policy value of network methods under real-world data constraints, while simultaneously providing empirical justification for investing in more systematic network data collection infrastructure. Future research should investigate different systems and make use of more complete datasets.

Additionally, studying how shocks propagate through these networks is vital for assessing the systemic stability of economic systems. A promising direction for future work is the joint analysis of credit and supply chain networks as a multilayered structure, enabling a fuller understanding of the interdependencies that shape firm behavior and systemic risk.

Ultimately, bridging ecological thinking and economic network analysis may offer valuable tools for anticipating, managing, and mitigating systemic risks in an increasingly interconnected world.

## Supplementary Material

pgag170_Supplementary_Data

## Data Availability

The raw data underlying this study are proprietary and cannot be publicly shared. Questions related to data can be addressed to Victoria Landaberry (mlandaberry@bcu.gub.uy)

## Notes

a Firms in the primary activities, real estate, and financial intermediation sectors appear only as debtors or creditors of the surveyed firms.
