# Supplementary material for: DebtStreamness: an ecological approach to credit flows in interfirm networks
Source: PNAS Nexus. 2026 May 18;5(6):pgag170. doi: 10.1093/pnasnexus/pgag170 (PMC13237432; doi:10.1093/pnasnexus/pgag170)
Supplement: pgag170_Supplementary_Data [file pgag170_supplementary_data.pdf]

PNAS Nexus, 2026, , 1–4
DOI added during production
Published: Date added during production
Supplementary Information

PNAS NEXUS

DebtStreamness: An Ecological Approach to Credit Flows in Inter-Firm Networks

Anahí Rodríguez-Martínez,[1] Silvia Bartolucci,[1] Francesco Caravelli,[2] Victoria Landaberry,[3] Pierpaolo Vivo[4] and Fabio Caccioli[1,5,*]

[1]Department of Computer Science, University College London, Gower Street, London, WC1E 6EA, United Kingdom
[2]Theoretical Division, Los Alamos National Laboratory, Los Alamos, 87545, New Mexico, USA
[3]Banco Central del Uruguay, 777 Diagonal J.P. Fabini. Montevideo, 11100, Uruguay
[4]Department of Mathematics, King's College London, Strand, London, WC2R 2LS, United Kingdom
[5]Systemic Risk Centre, London School of Economics and Political Science, Houghton Street, London, WC2A 2AE, United Kingdom
*To whom correspondence should be addressed: f.caccioli@ucl.ac.uk

DebtStreamness derivation

DebtStreamness (DS_i) is equivalent to the notion of *downstreamness* in the economic analysis of Input/Output system (3; 2; 1), this time computed within the inter-firm network using financial institutions as reference point:

$$DS_i = 1 + \sum_j A_{ij} DS_j \; . \tag{1}$$

DS_i represents firm i's average downstream position from financial institutions, which act as the initial lending node in the inter-firm credit network within the economy. Specifically, Eq. (1) defines DS_i for firm i as the value of the gross debt of firm i, positioned downstream (in the final stage of debt within the production process) with respect to financial institutions, which are positioned upstream and serve as the initial nodes. Let $\ell_{ij} = L_{ij}/D_j$ represent the fraction of firm j's debt that flows to firm i. Then, the DebtStreamness of firm i is given by:

$$DS_i = \frac{B_i}{D_i} + 2\sum_j \frac{\ell_{ij} B_j}{D_i} + 3\sum_{jk} \frac{\ell_{ik}\ell_{kj} B_j}{D_i} + \dots \; . \tag{2}$$

By re-summing (2) explicitly, we can rewrite it as

$$DS_i = \frac{\left[(\mathbb{1} - \mathcal{L})^{-2}\vec{B}\right]_i}{D_i} \; , \tag{3}$$

where $[\cdot]_i$ denotes the ith entry of the vector, $\mathcal{L} = (\ell_{ij})$, and $\vec{B}$ is the vector of the amounts borrowed by firms i from financial institutions. For each firm also the following accounting identity holds:

$$D_i = B_i + \sum_{j=1}^{N} L_{ij} \; . \tag{4}$$

Inserting now Eq. (4), which can be rewritten as

$$D_i = B_i + \sum_{j=1}^{N} L_{ij} = B_i + \sum_{j=1}^{N} \ell_{ij} D_j \Rightarrow \vec{D} = (\mathbb{1} - \mathcal{L})^{-1}\vec{B} \; , \tag{5}$$

into Eq. (3), we have the following

$$DS_i = \frac{\left[(\mathbb{1} - \mathcal{L})^{-1}\vec{D}\right]_i}{D_i} \tag{6}$$

where $\vec{D}$ is the $N \times 1$ vector representing the total amount of debt of firms.

Introducing the matrix

$$\Delta = \begin{pmatrix} D_1 & 0 & \cdots & 0 \\ 0 & D_2 & \cdots & 0 \\ \vdots & \vdots & \ddots & \vdots \\ 0 & 0 & \cdots & D_N \end{pmatrix} , \tag{7}$$

and recalling that $\ell_{ij} = L_{ij}/D_j$ and $A_{ij} = L_{ij}/D_i$ which implies $\mathcal{L} = L\Delta^{-1}$, $A = \Delta^{-1}L$ and therefore $\mathcal{L} = \Delta A \Delta^{-1}$, we can write from (6)

$$\vec{DS} = \Delta^{-1}(\mathbb{1} - \Delta A \Delta^{-1})^{-1}\Delta\vec{1} \; . \tag{8}$$

This expression can be further simplified by writing $\mathbb{1} = \Delta\Delta^{-1}$ and after simple manipulations as

$$\vec{DS} = (\mathbb{1} - A)^{-1}\vec{1} \; . \tag{9}$$

Here, $(\mathbb{1} - A)^{-1}$ is the analog of the Leontief-inverse matrix in Input-Output economics. We recall that the matrix A has elements $A_{ij} = L_{ij}/D_i$, each representing the share of the borrowing by firm j from firm i divided by the total debt of firm i (input-upstream matrix), while $\vec{1}$ is the vector $N \times 1$ of ones.

Effect of cycles on DebtStreamness

To illustrate the effect of loops on DebtStreamness, let us consider a simple stylized network of three firms.

Draft

© The Author(s) 2026. Copyright and licence statements to be updated by the publisher during production.

DebtStreamness is defined as the solution of

$$\overrightarrow{DS} = \overrightarrow{1} + A\,\overrightarrow{DS}\,,$$

which implies

$$\overrightarrow{DS} = (\mathbb{I} - A)^{-1}\overrightarrow{1}\,.$$

First, let us consider the matrix

$$A^{(1)} = \begin{pmatrix} 0 & 0 & 0 \\ 1 & 0 & 0 \\ 0 & 1 & 0 \end{pmatrix}\,,$$

which corresponds to a simple credit chain in which firm 1 borrows directly from the external financial sector, firm 2 borrows from firm 1, and firm 3 borrows from firm 2. In this case,

$$\overrightarrow{DS} = (I - A^{(1)})^{-1}\overrightarrow{1} = \begin{pmatrix} 1 \\ 2 \\ 3 \end{pmatrix},$$

so DebtStreamness coincides with the layering of firms along the borrowing chain.

Now, let us consider the matrix

$$A^{(2)} = \begin{pmatrix} 0 & \ell & 0 \\ 1 & 0 & 0 \\ 0 & 1 & 0 \end{pmatrix}\,,$$

where firm 1 borrows a fraction ℓ from firm 2 (and the remaining share $1-\ell$ from the external financial sector), firm 2 borrows from firm 1, and firm 3 borrows from firm 2. Firms 1 and 2 therefore form a credit loop.

Solving for DebtStreamness gives

$$\overrightarrow{DS} = \begin{pmatrix} \dfrac{1+\ell}{1-\ell} \\ \dfrac{2}{1-\ell} \\ \dfrac{3-\ell}{1-\ell} \end{pmatrix}.$$

In the acyclic case, DebtStreamness behaves as a distance-like measure from the external funding source. However, when a loop is present, credit can circulate repeatedly between firms 1 and 2 before reaching firm 3. This generates an amplification effect proportional to the factor $1/(1-\ell)$. The amplification becomes stronger the larger is the share of borrowing occurring within the network (i.e., the greater is ℓ), corresponding to a smaller fraction of direct borrowing from the external financial sector. Hence, DebtStreamness can be interpreted as a distance-like measure from external funding sources that also accounts for amplification generated by the recirculation of credit within credit cycles.

Further analyses

Data

The initial inter-firm credit network comprises 1,072 firms; however, before computing DebtStreamness, some firms are excluded from the sample, as detailed below, to guarantee the internal consistency of the measure. Figure 1 illustrates the different configurations of firms that are excluded from the computation of DebtStreamness (DS). Specifically, we remove firms with zero total debt, i.e., firms that do not borrow either from financial institutions or from other firms in the inter-firm credit network. Although these firms may appear in the database because they extend credit to other firms, they have no incoming credit paths and therefore no well-defined position relative to the primary credit originators (financial institutions). Since DebtStreamness measures the average distance from financial institutions across all possible borrowing paths, nodes without incoming debt links cannot be assigned a meaningful DS value. From a network perspective, these firms correspond to upstream pure lenders or isolated nodes in terms of borrowing relationships.

Figure 1 Schematic situation of firms not included in DS measure (red cross marks). This diagram shows nodes with zero debt that we are not taking into account in DS measure. This means that these firms are not borrowing from financial institutions and are not borrowing from other firms. They are included in the database because they lend to other firms, but they are disconnected or have no relationship with another firm or bank in terms of borrowing. This scheme illustrates these different cases.

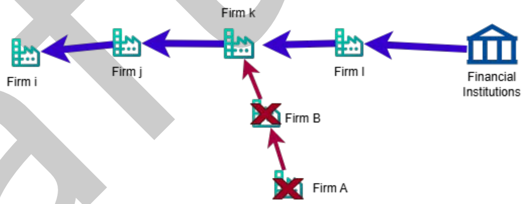

Although our analysis relies on data gathered for 2018 only, extensive empirical evidence indicates strong persistence in underlying buyer–supplier relationships. (4) show that approximately 80% of relationships survived the 2008–2009 trade collapse, while multi-country studies report monthly persistence rates near 95%, corresponding to a half-life of roughly 13 months (6; 5; 7). This structural stability supports the validity of our snapshot approach, as the core relational features of production networks remain largely stable over policy-relevant horizons. This framework enables the identification of systemic vulnerabilities and potential shock-propagation pathways that are unobservable in non-network settings.

For each firm in our dataset we observe the total volume of inter-firm borrowing F_i, but only the amounts associated with its top three creditors are explicitly reported. On average, these top creditors account for approximately 50% of a firm's total inter-firm credit. The remaining portion, defined as the residual

$$R_i = F_i - \sum_j L_{ij}^{\text{top}}\,, \qquad (10)$$

represents credit relationships that are known to exist in aggregate but for which the specific counterparties are unobserved. Figure 2 illustrates the magnitude of the residual inter-firm credit component. The top panel compares total inter-firm credit with this residual component, while the bottom panel shows the distribution of residual exposures. The evidence indicates that the residual primarily consists of

Draft

numerous smaller credit relationships rather than large missing exposures, suggesting that the dominant credit flows shaping firms' positions in the credit hierarchy are already captured by the observed top-creditor network.

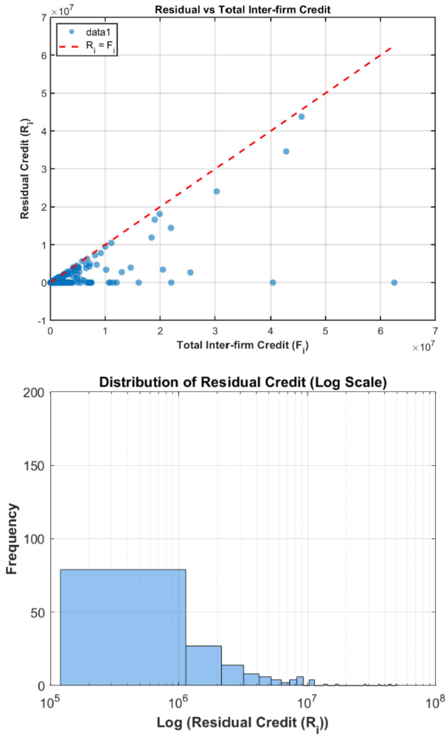

Figure 2 Residual and total inter-firm credit. Residual vs. total inter-firm credit and histogram of residual credit. **Top panel**: R_i vs F_i. **Bottom panel**: Histogram log residual credit.

Firms' Credit Score Analysis

In Figure 3, we report the share of firms within each credit risk group whose DebtStreamness (DS) exceeds a given threshold. The available credit score categories are $1C$, $2A$, $2B$, 3, 4, and 5, where $1C$ represents debtors with strong repayment capacity and 5 refers to unrecoverable debtors. To examine how network position varies with credit risk, we compute the fraction of firms whose DS exceeds four thresholds (DS $>$ 1.5, 2.0, 2.5, 3.0). High-risk firms tend to occupy more peripheral positions in the credit network. Across all thresholds, the high-risk group (scores 4 and 5) exhibits substantially larger exceedance fractions than the low- or mid-risk groups. At DS $>$ 1.5, nearly 43% of high-risk firms lie above this level, compared with 12% and 9% for the low and mid groups, respectively. At DS $>$ 2.0, the gap remains large (28.6% vs. 6.9% and 2.8%), indicating that the result is not driven by the choice of a single threshold. The low- and mid-risk groups behave similarly, with shares that remain close in magnitude across all thresholds; the mid group even falls slightly below the low group at DS $>$ 2.0 and above. Overall, the figure shows that deteriorating creditworthiness is associated with higher DS values, suggesting that riskier firms occupy more peripheral positions in the credit network and are consequently more exposed to indirect disruptions propagating through intermediation chains.

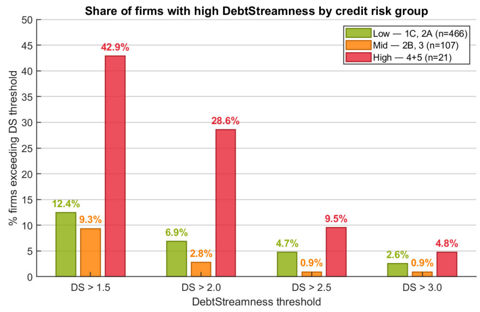

Figure 3 DebtStreamness distribution for each credit score. High-risk firms are significantly more likely to exhibit higher DS values than low- or mid-risk firms.

Draft

Network centrality

We compute the correlations between DS and network centrality measures, including degree, strength, eigenvector, betweenness, closeness centrality, PageRank, and a simplified DebtRank measure. The results are presented in Figure 4 (correlation matrix). Our analysis reveals that DS shows modest correlations with most traditional centrality measures ($|\rho| = 0.32$–0.74), with the strongest relationships observed for in-strength ($\rho = 0.74$) and degree measures ($\rho = 0.69$–0.72). Importantly, correlations with betweenness ($\rho = 0.63$) and eigenvector centrality ($\rho = 0.37$–0.42) are only moderate, demonstrating that DS captures a distinct dimension of network position beyond general topological centrality. We find a strong negative correlation with bank borrowing ($\rho =$ -0.68), validating that firms relying on direct financial sector access have lower DS. The high Spearman correlation with DebtRank ($\rho = 0.94$) indicates strong agreement in ranking systemically important firms, though with different conceptual foundations, DebtRank measures contagion potential while DS measures structural position in credit chains. These results confirm that DS provides complementary information to existing measures: while traditional centrality identifies generally well-connected or influential nodes, DS specifically captures credit intermediation depth and distance from primary financial sources.

Network reconstruction

To assess the sensitivity of our results to missing credit links, we reconstruct the network under alternative allocation schemes for the residual inter-firm credit in Eq. (10) (see Methods Section). In Fig. 5, we report the results for the sparse network reconstruction scenario, and the Input Output (IO) case, where missing links are filled proportionally to their respective IO production share. Across all reconstruction scenarios—including fully connected, sparse, and input-output–based imputations—DebtStreamness values remain almost unchanged, with rank correlations above 0.98. This confirms that our results are robust to the partial nature of the observed network.

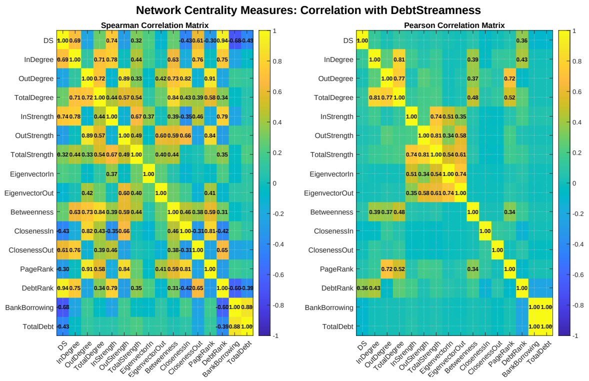

Figure 4 Correlation matrix. Spearman (Left Panel) and Pearson (Right Panel) correlation matrix between different network metrics (in- and out-degree/strength, pagerank), DebtRank, total borrowing and DebtStreamness.

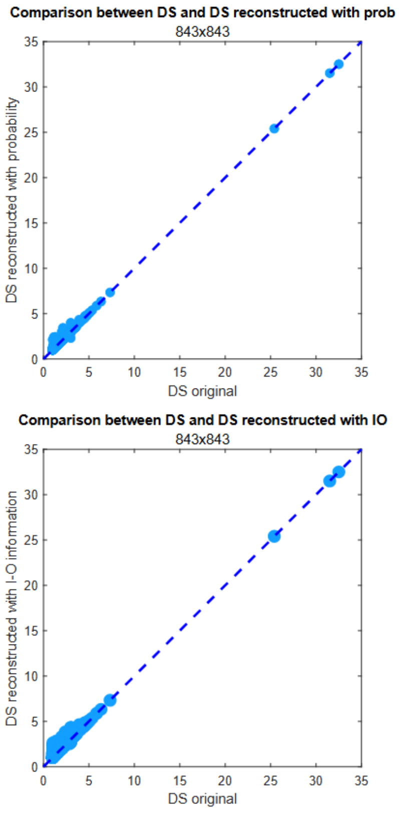

Figure 5 DebtStreamness in reconstructed networks. Comparison of DebtStreamness computed from the network with only top three creditors and the partially reconstructed networks. **Top panel**: comparison with partially reconstructed sparse network. **Bottom panel**: comparison with reconstructed network using Input-Output data and sector information.

References

1. Pol Antras, Davin Chor, Thibault Fally, and Russell Hillberry. Measuring the upstreamness of production and trade flows. *American Economic Review*, 102, 02 2012.
2. Silvia Bartolucci, Fabio Caccioli, Francesco Caravelli, and Pierpaolo Vivo. Correlation between upstreamness and downstreamness in random global value chains. *Journal of Economic Behavior & Organization*, 233:106945, 2025.
3. Silvia Bartolucci, Fabio Caccioli, Francesco Caravelli, and Pierpaolo Vivo. Upstreamness and downstreamness in input–output analysis from local and aggregate information. *Scientific Reports*, 15(1):2727, 2025.
4. Andrew B. Bernard, Andreas Moxnes, and Karen Helene Ulltveit-Moe. Two-sided heterogeneity and trade. *Review of Economics and Statistics*, 100(3):424–439, 2018.
5. András Borsos and Martin Stancsics. The hungarian production network: Persistence and dynamics. *Hungarian Statistical Review*, 3(2):3–28, 2020.
6. Emmanuel Dhyne, Glenn Magerman, and Stela Rubínová. The belgian production network 2002-2012. Working Paper 288, National Bank of Belgium, 2015.
7. Christian Diem, András Borsos, Tobias Reisch, János Kertész, and Stefan Thurner. Supply chain network rewiring dynamics at the firm-level. *arXiv preprint arXiv:2503.20594*, 2025. Available at: https://arxiv.org/abs/2503.20594.

Draft
